# A Bornean database of plant uses and their cultural contexts: Introducing BioCultBase\Borneo

**DOI:** 10.1016/j.dib.2024.110926

**Published:** 2024-09-14

**Authors:** E. Petter Axelsson, Daniel Lussetti, F. Merlin Franco

**Affiliations:** a Department of Wildlife, Fish and Environmental Studies, Swedish University of Agricultural Sciences, Sweden; b Department of Forest Ecology and Management, Swedish University of Agricultural Sciences, Sweden; c Institute of Asian Studies, Universiti Brunei Darussalam, Brunei Darussalam

**Keywords:** Human-nature interaction, Cultural ecosystem services, Cultural heritage, Sustainability, Local knowledge

## Abstract

Biocultural diversity is important for environmental justice, human wellbeing, and sustainable development. Yet it is threatened by landscape degradation and overexploitation. When species go extinct, there is a co-occurring loss of associated cultural elements, and marginalized cultures are the ones that suffer the most from these losses. Here, we present *BioCultBase/Bor*neo, a database of local uses of plants and their cultural contexts from the biologically and culturally hyper-diverse island of Borneo. The database has been developed from secondary data extracted from scientific literature, but is intended to be a live repository that welcomes contributions from academics, researchers and the general public. *BioCultBase/Bor*neo database currently covers 1319 confirmed plant species and plant parts used for 23 use categories. These uses are reported from 39 ethnic communities of Borneo, together representing at least 2242 unique ecocultural links. The ethnicities represented in the database cover 13 % of the 306 officially recognized ethnicities of Borneo. Developing the database further will enhance access to ecocultural data that can be used for developing policy and practises relevant for a broader range of peoples.

Specifications Table

**Table:** 

Subject	*Nature and Landscape Conservation*.
Specific subject area	*Biocultural diversity represents the diversity of life in all its manifestations: biological, cultural, and linguistic and is important for sustainable development*
Type of data	Table, Processed.
Data collection	*Relevant literature was compiled through a literature search in the Web of Knowledge database via the “topic” search feature including all databases available in the portal using a designated search string*
Data source location	*The data is stored with the Swedish National Data service*https://snd.se/en
Data accessibility	The data is stored with the Swedish National Data service Repository name: A Bornean database of plant uses and their cultural contexts: Introducing BioCultBase/Borneo https://snd.se/en/catalogue/dataset/preview/cee077c6-0501-41d3-b715-c5f1b4f618fe/1
Related research article	none

## 1 Value of the Data

• Policies highlight ecocultural information as important for environmental justice and for attaining sustainability goals.
• Transferring policies into practices require better understanding of the ecocultural assets of landscapes and regions.
• Ecocultural information compiled here can be used to inform conservation management.

## 2 Background

Sustainable development and environmental justice require a broad range of Nature's contribution to people to be incorporated into management of species and landscapes [1,2]. Cultural ecosystem services are now incorporated into policies. But realising affirmative practices require greater recognition of biocultural diversity (Box 1) [[3], [4], [5]].

Box 1 **Definition of Biocultural diversity**

“The diversity of life in all its manifestations: biological, cultural, and linguistic — which are interrelated (and possibly coevolved) within a complex socio-ecological adaptive system” Maffi 2007

Alt-text: Unlabelled box

Quintessential for achieving sustainability [1,2], biocultural diversity [[5], [6], [7]] is threatened by various forces including landscape degradation [4,8]. This is deleterious for local communities with intimate ties to landscapes, and that are on a perpetual struggle to have their worldviews acknowledged by modern economies [9]. Information on People-landscape links are typically scattered in the literature, hampering retrieval and use of ecocultural data.

In this background, we present the *BioCultBase/Bor*neo, a database of local uses of plants on the biologically and culturally hyper-diverse island of Borneo [10]. We envision that the database will enhance accessibility of ecocultural data for Borneo, thereby catalysing conservation strategies that encompass a wider range of culturally significant plants, from a broader range of cultures.

## 3 Data Description

Our data compilation focuses on the biologically and culturally diverse island of Borneo. Borneo is politically divided between Brunei, Indonesia, and Malaysia, and represents the largest land mass of the Sundaland biodiversity hotspot. As such, the island has rich and unique biodiversity, with a considerable portion of plants and animals being endemic. Borneo is also ecoculturally diverse with local communities belonging to 306 officially recognized native ethnicities (see below). The database is based on information recorded in scientific literature but is intended to be a dynamic repository that welcomes contributions from peers and the public.

**Fig. 1:**
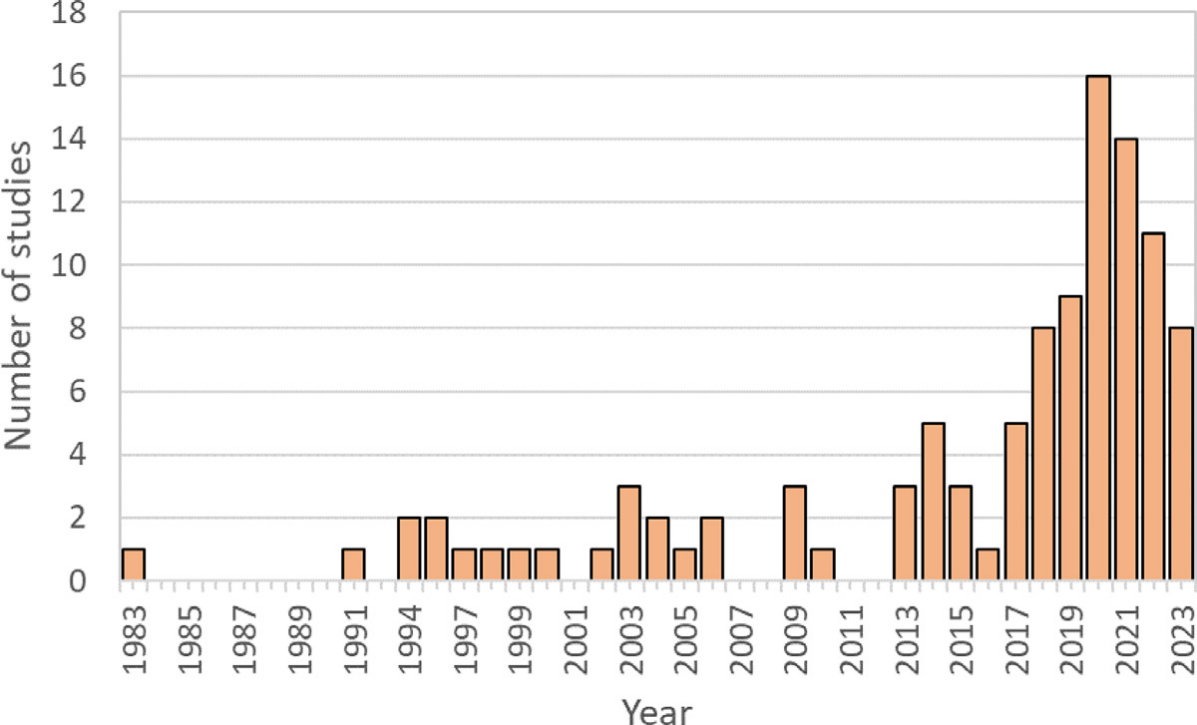
Number of studies that providing ecocultural information to the BioCultBase/Borneo database distributed per publication date.

The 106 articles identified through our literature search represent ecocultural information recorded over a period of 40 years, from 1983 to 2023 (Fig.1). Data include scientific and local plant names, plant parts used, and uses divided into 23 use categories, and ethnic identity of informants. Overall, the BioCultBase/Borneo includes ecocultural information for 1319 confirmed plant species to date, and 194 records for unconfirmed binomials. The data provided include 23 use categories and specifics (Table 1), as well as plant parts used. Cultural uses of plants is recorded from 39 of native ethnicities of Borneo, representing 13 % the 306 officially recognized ethnicities of Borneo. The database currently represents at least 2242 unique ecocultural links. The included files represent the database (BioCultBase_2024-08-22.tsv), original data sources (References.tsv) information on how the data was collected; search string (Appendix_1_search.txt), criteria and categories (Read me.txt).

**Table 1:** Twenty-two (23) use categories specified in the BioCultBase/Borneo database and their explanation.

Use categories	Explanation
Food	Plant parts used for consumption and in preparing food (fruits, leaves etc.)
Seasoning	Plant parts uses as spices, sweetener in food
Fuelwood	Plant parts used for heating (firewood, charcoal etc.)
Forage/Fodder	Vegetation used as forage/fodder for livestock
Wax	Plant species associated with wild bees that produce wax
Latex/Gum	Sap and other plant parts that can be used for producing latex or gum
Fabric/Fibre	Plant parts used to produce fibre and different types of fabric
Dye	Plant parts used for colouring/dying
Construction	Basic plant parts used for construction of houses and infrastructure (logs, beams, bark and fronds etc.)
Tools	Plant parts used to produce different kind of tools
Basketry	Plant parts used for making baskets
Furniture	Wood and other plant parts used for furniture. Often more specific than construction
Handicraft	Wood and other plant parts used for handicrafts. Often more specific than construction
Boat building	Wood and other plant parts used for building boats and ores. Often more specific than construction.
Musical instrument	Plant parts used for construction of musical instruments (wood, bamboo etc.)
Cosmetic	Plant parts yielding ingredientscosmetics
Traditional medicine	Plant parts used for preparing traditional medicinal formulations
Veterinary medicine	Plant parts used for treating diseases of livestock
Biological control	Plant parts used as biological control (mosquito repellent, crop protection etc.)
Wildlife	Plants with special association with wildlife
Poison	Plant part used to produce poison (poison to catch fish etc.)
Ornamental	Plants or plant parts used for aesthetic purposes
Ceremonial/spiritual	Plants or plant parts with spiritual or ceremonial significance. Ceremonial decorations, incense that cast away evil spirits, totem species etc.
Other	Other uses that do not apply to any of the above

## 4 Experimental Design, Materials and Methods

To build the database, we conducted a literature search in the web of knowledge database via the “topic” search feature including all databases available in the portal using a search string composed of two main components. The first component limited our search to the focal region of Borneo by listing Borneo(including sub regions). To this first component, we also added a comprehensive list of 306 indigenous ethnic groups for Brunei, Sabah, and Sarawak (Malaysian Borneo), and Kalimantan (Indonesian Borneo). For ethnicities of Sabah, we included 69 distinct ethnicities following Combrink et al. [11]. For Brunei, we listed eight ethnicities: Malay, Tutong, Kedayan, Dusun, Murut, Bisaya, Belait, and Iban. The first seven of these communities are recognised by the Brunei Government as indigenous to Brunei [12]; Iban are indigenous to Borneo, but not politically recognised as indigenous to Brunei [6]. For Sarawak, we included the 24 ethnicities listed on the Sarawak government official portal [13], and the twelve indigenous groups that were recently recognised as native ethnicities by the Sarawak State Legislative Assembly [14]. For Kalimantan, we included the 193 ethnicities listed in Na'im and Syaputra [15]; we also consulted Arifin et al. [16]. In the second component, we listed a selection of search terms related to cultural uses and relevant synonyms. The full search string can be found in appendix 1 (see below). The search that was conducted on April 25 generated 1289 research articles whose titles and abstracts was scanned by two independent reviewers. Articles were deemed potentially relevant if they addressed questions in relation to cultural uses of plants by local cultures of Borneo. Articles addressing phytochemical properties of plant parts were also retained at this stage if their titles and abstract indicated local plant use. Through this, we identified 393 potentially relevant articles which we scrutinized for data relevant to our research topic. To build our database, we then extracted information on cultural uses including plant parts used, and ethnic identities of users. In total, there were 106 articles providing data to the database.

## Limitations

The compiled data are limited to data from scientific publications written in English. Hence, it does not include reports in local languages or information recorded in books.

## Ethics Statement

Authors have read and followed the ethical requirements for publication in Data in Brief and confirm that the current work does not involve human subjects, animal experiments, or any data collected from social media platforms.

## CRediT authorship contribution statement

**E. Petter Axelsson:** Conceptualization, Methodology, Formal analysis, Investigation, Data curation, Writing – original draft, Writing – review & editing, Visualization, Project administration, Funding acquisition. **Daniel Lussetti:** Conceptualization, Methodology, Writing – review & editing. **F. Merlin Franco:** Conceptualization, Methodology, Writing – review & editing.

## Data Availability

BioCultBase/Borneo(Original data) (Swedish National Data service).

## Appendix Supplementary materials

Image, application 1
