# Supplementary material for: A Bornean database of plant uses and their cultural contexts: Introducing BioCultBase\Borneo
Source: Data Brief. 2024 Sep 14;57:110926. doi: 10.1016/j.dib.2024.110926 (PMC11440315; doi:10.1016/j.dib.2024.110926)
Supplement: Supplementary file 1 [file mmc1.docx]

Appendix 1: Search string used to identify peer review papers of relevance to plant uses by officially recognized ethnic groups of Borneo

(Borneo or Sabah or Brunei or Sarawak or Kalimantan or BAJAU Or Banggi or Baukan or Begak or BISAYA or BONGGI or BOOKAN or Brunei or BUGIS or “Bulud Upi” or “CENTRAL DUSUN” or “COASTAL KADAZAN” or “COCOS ISLANDS MALAY” or DUMPAS or Dusun or “Eastern Kadazan” or GANA or IDA'AN or Illanun or IRANUN or Kadazan or KADAZANDUSUN or KAGAYAN or KALABAKAN or KALABUAN or KEDAYAN or KENINGAU MURUT or KIMARAGANG or Kolod or KUIJAU or “Labuk Kadazan” or “LABUK-KINABATANGAN KADAZAN” or LOBU or LOTUD or LUNDAYEH or MAKIANG or MANGKAAK or MINOKOK or MURUT or Nabay or OKOLOD or PAITAN or PALUAN or PAPAR or Rumanau or RUNGUS or “SELUNGAI MURUT” or SEMBAKUNG or SERUDUNG or SINABU' or “SOUTHERN SAMA” or SULUK or Sumambu or SUNGAI or TAGAL or TATANA' or Tausug or TEMPASUK DUSUN or TENGARA or TIDUNG or TIMUGON or TINAGAS or TOBILUNG or TOMBONUO or Tuaran Dusun or UBIAN or Ulun-no-Bokan or “UPPER KINABATANGAN” or “WEST COAST BAJAU” or Malay or Tutong or Kedayan or Dusun or Murut or Bisaya or Belait or Iban or Iban or Malay or Bidayuh or Melanau or Kayan or Kenyah or Lun Bawang or Penan or Kelabit or Kedayan or Bisaya or Berawan or Lahanan or Sekapan or Kejaman or Punan or Baketan or Ukit or Sihan or Tagal or Tabun or Saban or Lisum or Longkiput or Bagatan or Bakong or Bemali or Berawan or Dali or Lakiput or “Jatti Miriek” or Narom or Sa’ban or Tatau or Tring or Vaie or Dayak or Banjar or bai or Tidung or Tingalan or Tudung or Abal or Ahe or Anas or Toi or Apalin or Palin or “Ata Kiwan” or Auheng or Ayus or Bentian or Karau or Lemper or Leo Arak or Bentian or Karau or Lemper or “Leo Arak” or Badeng or Bahau or Baka or “Bakung metulang” or Balangan or Banjau or Bantai or Bantian or “Bara dia” or Barangas or Basap or Bawo or Beketan or Benuak or Beraki or Berangas or “Berau ormerau” or Berusu or “Bugis Pagatan” or Bukat or Buket or Bukut or Ukit or Bulungan or murut or Bungan or Busang or Cika or dawam or rawan or “dayak Bawo” or mangkatip or Taboyan or “dusun deyah” or empran or gaat or gaura or Harakit or Huang Tering or “Hulu Banyu” or humba or sumba or “Tau humba” or Kajang or Kejin or Kenyah or Kanowit or Karehan or Kriau or Katibas or Katingan or ngaju or Kencing or Kendayan or Klemantan or Kutai or “Lapo Bakung” or “Lapo Bem” or “Lapo Ke” or “Lapo Kulit” or “Lapo maut” or “Lapo ngibun” or “Lapo Timai” or “Lapo Tukung” or Lawangan or “Lepo Bakung” or “Lepo Jalan” or “Lepo Tukung” or “Lepo Tepu” or “Lepo mant” or “Lepo Tau” or “Long gelat” or Paka or “Long Paka” or Lundayeh or maanyan or “maanyan Benua Lima” or “maanyan Paju Lima” or “maanyan dayu” or “maanyan Paju epat” or “maanyan Paju sepuluh” or “maanyan Paku” or malang or “mangku Anam” or nyumit or Pauk or Purui or “singa rasi” or Tungku or medan or modang or medang or merab or murung or ngalampa or nganayath or noenleni or oheng or ohong or “oloh Kantu'” or “oloh masih” or “orang gunung” or Pagatan or Paku or Pasir or Penihing or Pitap or “rai hawu” or Sawu or rangga or remucles or Sagai or Samihim or Sani or saqi or sarbas or saribas or sebayau or segayi or sekadau or sekayang or senunang or seputan or seru or serul or srul or “siak murung” or “siang murung” or siang or skrang or sului or suntung or Tabuyan or Tagel or Tagol or Tamuan or Tana' or Telaga or Timai or Tomun or Tou or Touk or Tukung or Tumbit or Tungui or Tunjung or “Ulu Batang Ali” or “Umaq Alim” or “Umaq Baka” or “Umaq Bakaq” or “Umaq Baqaq” or “Umaq Jalan” or “Umaq Lasan” or “Umaq Pramuka” or “Umaq suling” or “Umaq Tau” or “Umaq Badang” or “Umaq Kulit” or “Umaq Lokan” or “Umaq Lasung” or “Umaq Leken” or “Umaq naving” or “Umaq Paku” or “Umaq Pliau” or “Umaq PugungPuh” or “undang Sanang” or undup or warukin or Menui or “Suku-suku lainnya di Kalimantan”) AND (“biocultural” OR “bio-cultural” OR "cultural keystone species" OR "CKS" OR “Cultural species” OR "Ethnobotany" OR "Ethnomedicine" OR “traditional medicine” OR “folk medicine” OR "Non timber forest products" OR NTFP OR "indigenous ecological knowledge" OR “indigenous knowledge” OR “traditional knowledge” OR “Local knowledge” OR “folk knowledge” OR "Forest garden" OR "indigenous agroforestry" OR simpukng OR "edible fruit” OR “wild edibles” OR “customary land”)
